# Effects of panax notoginseng saponins on the osteogenic differentiation of rabbit bone mesenchymal stem cells through TGF-β1 signaling pathway

**DOI:** 10.1186/s12906-016-1304-9

**Published:** 2016-08-26

**Authors:** Yan Wang, Xuanping Huang, Yiyao Tang, Haiyun Lin, Nuo Zhou

**Affiliations:** College & Hospital of Stomatology, Guangxi Medical University, 22, Shuangyong Road, Nanning, Guangxi China

**Keywords:** TGF-β1, Bone mesenchymal stem cells, Panax notoginseng saponins, Osteogenic differentiation

## Abstract

**Background:**

Panax Notoginseng is a well-known Chinese medicinal herb which has been used in China for treatment of bone fracture for hundreds of years. However, the specific biological mechanisms of osteogenic effect of PNS are not well understood.

**Methods:**

In this study, newborn rabbit BMSCs were isolated, and then identified by the positive expression rates of cell surface markers, including CD29, CD45 and HLA-DR, which were detected by flow cytometry(FCM). After the lentivirus-induced cell model of TGF-β1 gene silencing was established, the interference efficiency was tested by q-PCR and Western blot, and the growth curve of silencing cells was drawn by MTT so as to grasp the growth rhythm of silencing cells. In the alizarin red-staining experiment, the effect of 100 mg/L PNS on the activity of intracellular ALP of TGF-β1 gene silencing BMSCs was detected, so as to observe the effect of 100 mg/L PNS on the formation of calcium nodes of gene silencing BMSCs.

**Results:**

By separating rabbit BMSCs, the lentivirus-induced cell model of TGF-β1 gene silencing was established. Both TGF-β1 mRNA and protein expression were restrained significantly, and the target gene kept silence stably via the verification of q-PCR and Western blot; there was no significant differences of the growth curve between RNAi cells and normal cells; the activity of intracellular APL in 100 mg RNAi group was obviously lower than that in 100 mg group (*p* < 0.05), but higher than that in the normal group; in the alizarin red-staining experiment, it focused on the effects of PNS on the formation of calcium nodes of gene silencing BMSCs, which showed that calcium nodes could be formed in 100 mg RNAi group but its quantity was lower than that of 100 mg group (*p* < 0.05).

**Conclusions:**

It was shown that silencing TGF-β1 gene could interrupt the osteogenic effects of PNS. PNS may have a promoting effect on osteogenic differentiation of rabbits’ BMSCs in vitro by up-regulating the gene expression of TGF-β1.

## Background

Panax Notoginseng known as San Qi or Tian Qi is a well-known traditional Chinese medicinal herb which has been used in China since ancient time [1]. The key bioactive component of Panax Notoginseng is Panax Notoginseng Saponins (PNS) [2]. Many studies have been reported that PNS might be a potential treatment choice for various diseases. Recent studies have found that the hypoglycemic and anti-obesity properties of PNS may play an important role in the treatment of diabetes [3]. PNS injection has been reported may provide another choice for patients with angina pectoris (AP), although application of PNS alone showed no significant better or worse effect on AP patients, evidence showed PNS combined with traditional western medicine was a better treatment option for AP patient in improving patients’ clinical symptoms [4]. Nan et al. [5] found that PNS could inhibit phenotype switching of vascular smooth muscle cells (VSMCs) induced by Notch3 silencing in a dose-dependent manner, which might provide new evidence for searching effective drug for amending stability of atherosclerotic disease. PNS was also reported could attenuate colitis in azoxymethane/dextran sulfate sodium (DSS)-induced colitis mouse model [6].

In China, PNS has been applied in treatment of promoting bone fracture healing for hundreds of years [7]. Previous studies has been reported that PNS could stimulate alkaline phosphatase activities and increase the number of osteoblasts in vitro, promote the proliferation of bone marrow mesenchymal stromal cells [8], and is effective in promoting blood circulation to remove stasis [9]. Recent studies suggest that PNS may be a potential therapeutic methods for treating bone nonunion, osteoporosis and osteonecrosis [10]. However, the specific biological mechanisms of osteogenic effect of PNS are not well understood. This study focused on the relationship between osteogenesis of PNS and Transforming Growth Factor-Beta 1 (TGF-β1) signaling pathway, so as to reveal the gene targets of pharmacological action of osteogenesis of panax notoginseng at the molecular level. Thus, it may provide a research foundation for the exploration on the application of new Chinese medicines for various bone-repairs.

## Methods

### Experimental animals

This study was approved by the animal ethics committee of Guangxi Medical University. Healthy 1 to 2-day newborn New Zealand rabbits (without gender limitations) were supplied by the Experimental Animal Center of Guangxi Medical University in Nanning, China (License No.: SCXK GUI 2009–0002).

### Reagents and instruments

Reagents and instruments were showed as following, including PNS (freeze-drying Xue Shuan Tong injection, Wuzhou Pharmaceutical Group Co. Ltd, Wuzhou, China, No.Z20025652, 150 mg/bottle); L-DMEM culture medium and fetal bovine serum (FBS) (GE Healthcare HyClone™ Cell Culture Co., Logan, USA); 0.25 % trypsin (Shanghai Bi Yun Tian Biotechnological Co. Ltd, Shanghai, China); osteogenesis-induced liquid (Nanning Weierkai Biotechnological Co. Ltd, Nanning, China); Methylthiazoletetrazolium (MTT) assay and percoll separating medium (Sigma-Aldrich Co., St. Louis, USA); rabbit anti-human CD29 polyclonal antibody (FITC) (Beijing Biosynthesis Biotechnological Co. Ltd, Beijing, China); rat anti-human and rabbit CD45 monoclonal antibody (FITC) (AbD Serotec Co., Oxford, UK); rat anti-human and rabbit HLA-DR monoclonal antibody(FITC) (Bio-Rad Co., Hercules, USA); rat anti-human IgG1(FITC) and IgG2b(FITC) (Lianke Biotechnological Co., Hangzhou, China); total RNA-extracted kit (Corning Incorporated Co., New York, USA); reverse transcription kit (TaKaRa Co., Tokyo, Japan); real-time PCR primer (Shanghai Bioengineering Co. Ltd, Shanghai, China); DAB kit (Beijing Zhongshan Biotechnological Co. Ltd, Beijing, China); rat anti-rabbit TGF beta 1 antibody (Novus Biologicals Co., Littleton, USA); goat anti-rabbit IgG (Bio-Techne Co., Minneapolis, USA); Alkaline Phosphatase(ALP) detection kit (Nanjing Jiancheng Biotechnological Co. Ltd, Nanjing, China); alizarin red kit (Shanghai SSS Reagent Co. Ltd, Shanghai, China); CO2 calorstat (Themo Forma Co., Waltham, USA); inverted aberration microscope (Zeiss Co., Oberkochen, Germany); PCR detection system (Bio-Rad Co., Hercules, USA).

### Isolation, culture and identification of rabbit bone marrow mesenchymal stem cells (BMSCs)

Following a protocol approved by the animal ethics committee of Guangxi Medical University, rabbit bone marrow were obtained from tibia and femur of new born New Zealand rabbit by flushing the marrow cavity using a 5 ml syringe filled with L-DMEM-medium (including 10 % FBS and 1 % penicillin-streptomycin) attached with a 23G needle. Then the medium with bone marrow was added into the centrifuge tube (pouring slowly along the tube wall and avoiding shaking) with isopyknic Percoll cell separation solution (1.073 g/ml). After 20-minute 2000 rpm centrifugalization, the nebulous liquid in the middle layer was carefully moved into a new centrifuge tube. And medium was added into the tube for 10-minute 1000 rpm centrifugalization. Then resuspended the cells and regulated the cell concentration to 5 × 10^5^/ml. Afterwards, the cells were collected and cultured in a 50 ml culture flask with L-DMEM-medium supplemented with 10 % FBS and 1 % penicillin-streptomycin at 37°Cin a 5%CO2 humidified incubator. The nonadherent cells were removed after 48 h and the medium was changed every 3 days. The cell passaging was achieved when the cell confluence reached to 80–90 %. After that, the growing status of cells was observed under the inverted aberration microscope. The monoclonal antibody, such as CD29, CD45 and HLA-DR, was added in 3rd passage of cells which grew in a good condition, and the FCM was applied to detect the positive rate of each surface marker in cultured cells.

### Construction of lentivirus-mediated siRNA interference cell model

On the basis of NCBI-GenBank (National Center for Biotechnology Information GenBank) information for TGF-β1 related gene sequence of New Zealand rabbit, we chose the relatively complete mRNA sequence (Oryctolagus cuniculus transforming growth factor, beta 1(TGF-β1), mRNA, NCBI Reference Sequence: XM_00272231312, 1164 bp), as the RNA interference target sequence. Nanning Weierkai Biotechnological Co. Ltd. was responsible for the synthesis of siRNA sequence, including the screening of optimal sequence (Lipofectamine^TM^ RNAi MAX transient transfection), the construction of TGF-β1 shRNA lentivirus vector (including ZsGreen green-fluorescent protein), the lentiviral packaging and titering and detection of the virus multiplicity of infection (MOI).

### The verification of silencing effect via q-PCR

The total RNA was extracted from 48-hour transfected BMSCs by Trizol method. Extracted RNA(2 μg) was converted into cDNA via reverse transcriptase and was put into the real-time q-PCR: primer TGF-β1-F: 5’ AAGTCGGCACAGCGTCTATA, TGF-β1-R: 5’ TTGCTGCATTTCTGGTACAG, amplified fragment 150 bp; β-actin-75 bp, β-actin-F: 5’ ACTGGAACGGTGAAGGTGACA, β-actin-R: 5’ TCGGCCACATTGCAGAACT; the reaction condition of PCR was showed as following: 50 °C 2 min; 95 °C 2 min; 95 °C 15 s, 60 °C 32 s, reading plates, 40 circulations; 60 °C-95 °C melting curve analysis. The RQ value should be calculated and repeated three times for each sample.

### The verification of silencing effect via Western blot

The total protein was extracted from 48-hour infected BMSCs by cluster-cracking reaction, and those protein samples were quantified via BCA method. 30 μg protein was transferred to PVDF membrane by SDS-PAGE electrophoresis. After Western blotting and development(compared with GAPDH), the sample was taken a picture via infrared imaging system. The gray value of plaques was analyzed and compared among groups.

### Measurement of transfected BMSCs proliferation via methylthiazoletetrazolium (MTT) assay

After two passages, the cultured nontransfected BMSCs, growing in a good condition, were seeded in 50 ml culture flasks(Corning Co.). When the confluence of cells reached 40 % in the flask bottom, the pre-diluted blank virus solution or virus solution was added into the culture flask. After culturing the cells in incubator at 37 °C in 5 % CO_2_ for 24 h, we added normal culture medium for cell amplification. Then, the transfected cells were seeded in 96-well culture plates. Comparing with normal cells cultured in the same density, the cell growth curves of the 1st, 3rd, 5th, 7th, 9th and 11th day were drew via MTT method, so as to observe the effect of gene silencing on BMSCs proliferation.

### Effects of PNS on intracellular ALP activities

With the density of 10^3^–10^4^/ml, BMSCs, growing in a good condition, were cultured in a 24-well plate. When the confluence of cells reached to 30 %, we divided the cells into five groups, including blank group, 100 mg group, blank virus group, RNAi group and 100 mg RNAi group. The blank virus group was transfected by blank virus diluents, and RNAi group and 100 mg RNAi group were transfected by virus diluents(silencing method) for 24 h. The medium was changed on the next day of all groups, and cells in 2nd and 5th group were also treated with PNS at 100 mg/L. After the 3rd, 5th and 7th day, the medium was eliminated, and each well was washed by 1 ml PBS once. Then, 500 μl TritonX-100 cell lysis solution was added for 40-minute ice-bath lysis. Under the instruction of ALP detection kit, the OD value of 30 μl lysis solution from each well was detected by ELISA in 520 nm wave length. Finally, the King Unit of ALP in each sample should be calculated in accordance with the formula.

### Effects of PNS on the formation of calcium nodes of gene silencing BMSCs via alizarin red-staining method

With the density of 10^3^–10^4^/ml, BMSCs, growing in a good condition, were cultured in the 6-well plate. When cells were occupied 30 % of the bottom, those wells were divided into six groups, including blank group, positive group, 100 mg group, blank virus group, RNAi group and 100 mg RNAi group. The blank virus group was infected by blank virus diluents and the last two groups by virus diluents(silencing method) for 24 h. Next day, the osteogenesis-induced solution was put into the positive group, and PNS 100 mg/L pure culture solution into both 100 mg group and 100 mg RNAi group, while the pure culture solution into the rest three groups. Each solution above was changed every 2 days to maintain the induction. After the next 21-day culture, the alizarin red was added into each well for staining under the instruction of kit. The quantity of calcium nodes should be calculated via 8 visual fields which were chose from each sample randomly under the microscope, so as to use both mean and standard deviation to make the comparison among those groups.

### Statistical analysis

Quantitative data were presented as mean + SD. Statistical significance was determined by two-tailed student’s *t*-test or one-way ANOVA followed by the LSD *t*-test for multiple comparisons. A *P*-value of 0.05 was considered statistically significant.

## Results

### Isolation, culture and identification of rabbit BMSCs

**Fig. 1:**
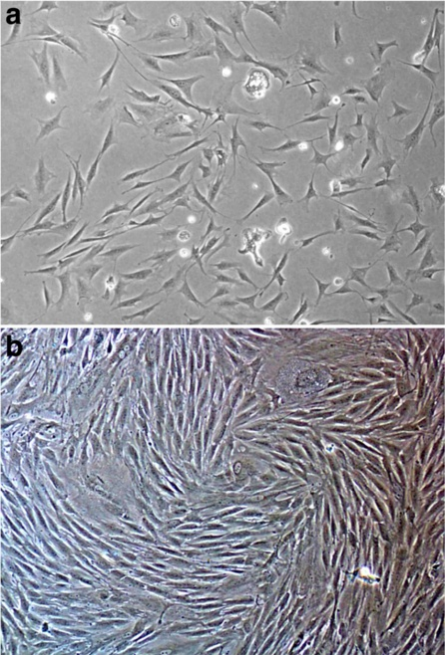
Subcultured BMSCs (P3) Cells reached 80–90 % confluence after 7 days of culturing 100× (**a**:3d **b**:7d)

**Fig. 2:**
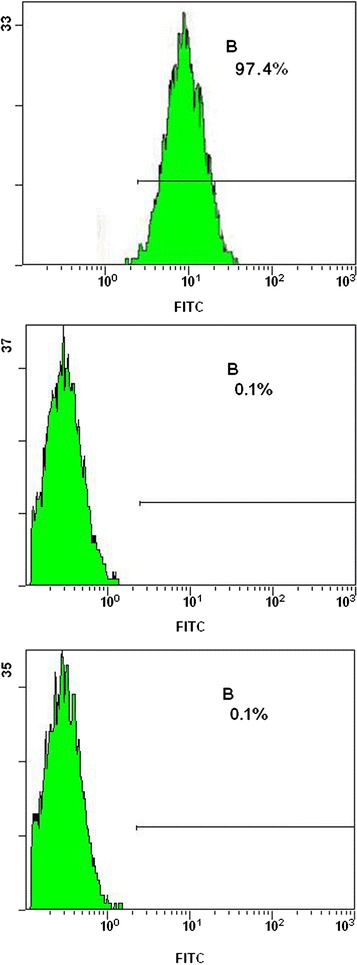
The expression of surface markers of BMSCs by FCM. Positive rate of surface marker: CD29: 97.4 %, CD45: less than 1 %, HLA-DR: less than 1 %

After the first medium change for primary cultures at 48 h, a large number of adherent cells with irregular shape were observed. Polygon-like tentacles were found on some of the cells. As the cell number increased rapidly, gradually formed cell colonies were observed. Most cell colonies were composed of cells with a characteristic spindle-like or long spindle-like shape, and arranged in a whorl-like array. Primary cultured cells reached 80 % confluence after 7–8 days of seeding. After 2–3 subcultures, cells reached 80–90 % confluence after 6–7 days of culturing (Fig. 1). The results of flow cytometry showed that the positive rate of P3 BMSCs’ surface marker CD29(integrin family member) was more than 95 %, while the positive rate of CD45(development phase marks of hemocytes) and HLA-DR(surface marks of fibroblasts) was less than 1 %(Fig. 2).

### RNA interfering BMSCs cell model

**Fig. 3:**
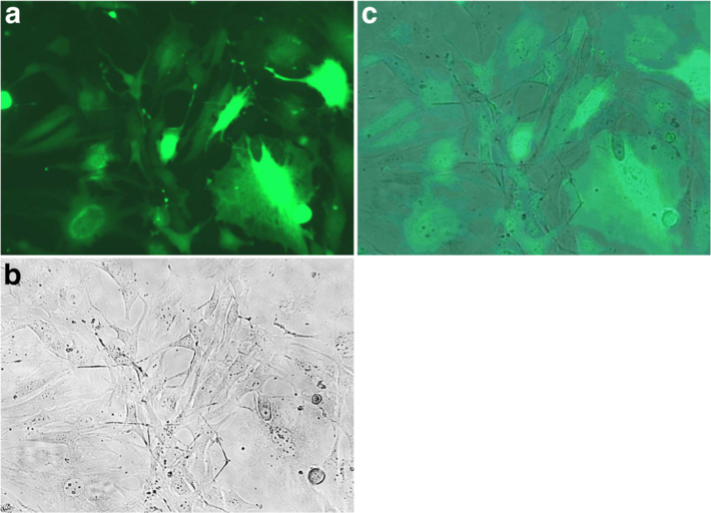
Observation of BMSCs after 48-hour virus infection 200× (**a**. fluorescence photo, **b**. normal photo, **c**. overlapping photo)

**Fig. 4:**
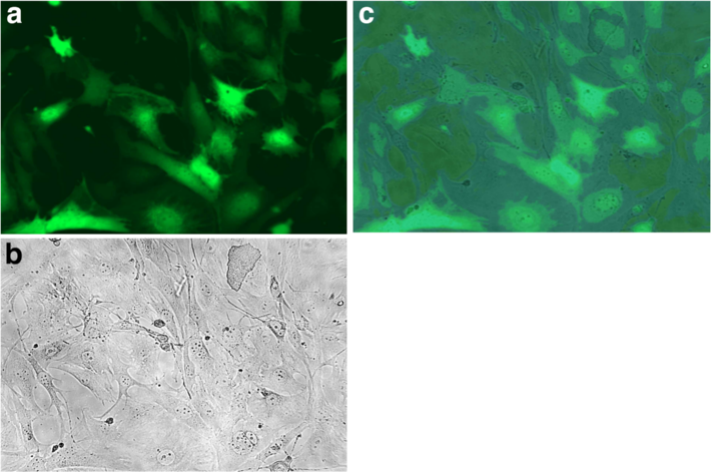
Observation of BMSCs after 72-hour virus infection 200× (**a**. fluorescence photo, **b**. normal photo, **c**. overlapping photo)

The interference efficiency of siRNA was detected and the optimal sequence(5’-3’) was screened as following: GCUUCAGAUCCACAGAGAAdTdT and antisense strand(AS) UUCUCUGUGGAGCUGAAGCdTdT. After the fluorescence observation, MOI was detected as 100 and the infection rate of cells was 80 % (Figs. 3 and 4).

### Results of TGF-β1 gene expression verification by real-time RT-PCR after RNAi

**Fig. 5:**
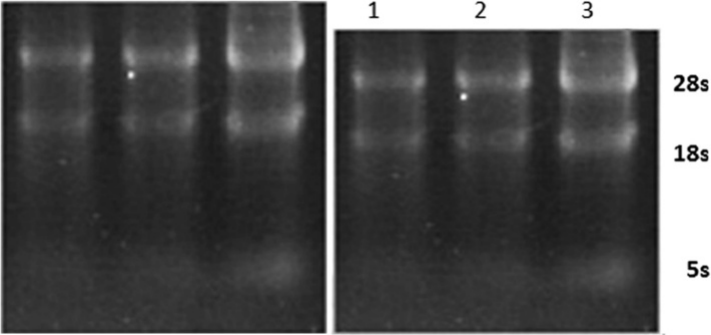
Electrophoretogram of extracted total RNA (1. normal cells, 2.blank virus-infected cells, 3. RNAi cells)

**Fig. 6:**
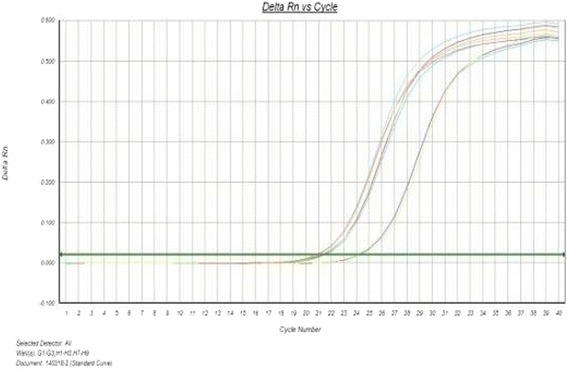
Amplification curve of TGF-β1 gene in each group

**Fig. 7:**
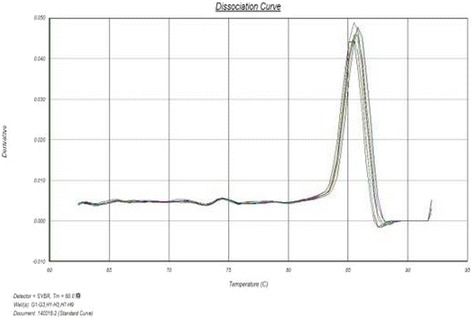
Melting curve of TGF-β1 gene in each group

**Fig. 8:**
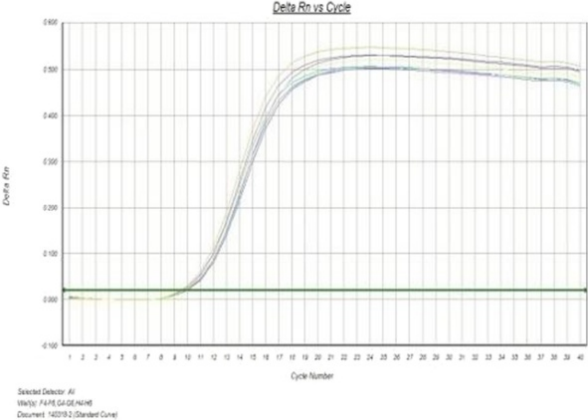
Amplification curve of β-actin in each group

**Fig. 9:**
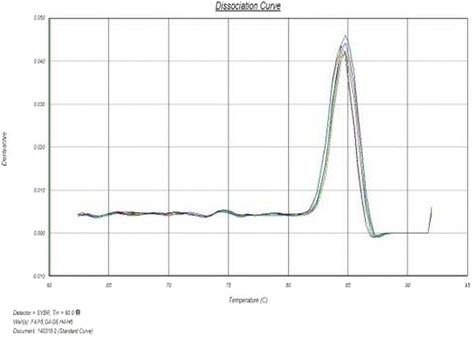
Melting curve of β-actin in each group

**Fig. 10:**
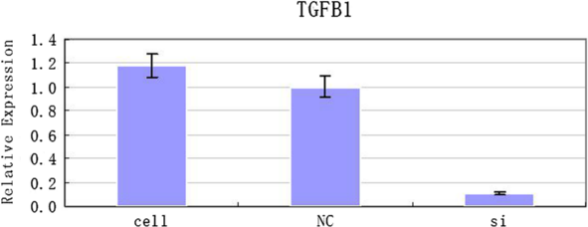
Comparison of TGF-β1 mRNA expression of normal group, blank virus group and siRNA group after virus-transfection

As shown by figures above (Figs. 5, 6, 7, 8, 9), the amplification curve and melting curve of each sample had repeatability, as well as both were smooth unimodal curves without irregular peaks. It was showed that both target genes and reference genes were amplified successfully. From the result of qRT-PCR (Fig. 10), the expression of TGF-β1mRNA of the RNAi group decreased significantly by comparing with the normal group. However, the expression of blank virus group was the same as the normal group. It suggested that siRNA-carried virus had an inhibitory effect on the gene expression of TGF-β1, while the blank virus had no effects on the expression of TGF-β1mRNA.

### Results of TGF-β1 gene expression verification by Western blot after RNAi

**Fig. 11:**
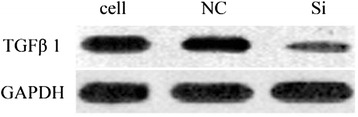
Western blot of TGF-β1 protein of normal group, blank virus group and RNAi group

According to the figure and table above, the virus transfection in RNAi group reduced the protein expression of TGF-β_1_ significantly by the comparison of the normal group; gene silencing was significant while there was no effect in the blank virus group (Fig. 11, Table. 1).

**Table 1:** Gray level results

Groups	Normal group	Blank Virus group	RNAi group
TGFβ1	16983.87	16853.84	4280.67
GAPDH	20169.29	22486.75	22750.20

### The growth curve of BMSCs after transfection

**Fig. 12:**
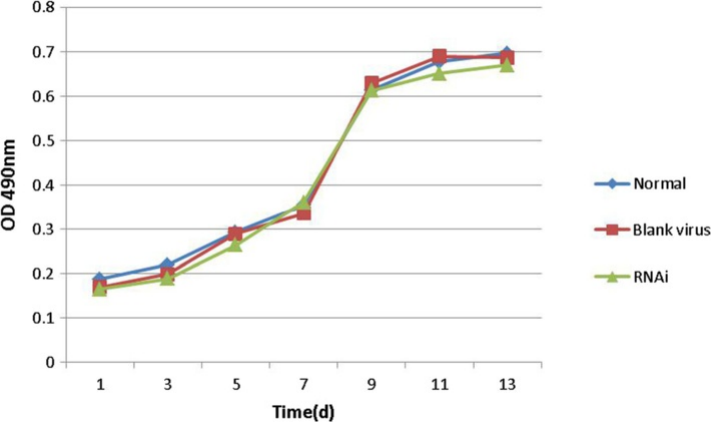
Growth curve of normal BMSCs and siRNA-transfected BMSCs

According to the figure above, after blank virus transfection and virus transfection, the growth curve of transfected cells was similar to that of normal cells, which showed that the transfected virus and gene silencing had little effects on cell proliferation (Fig. 12).

### The effect of PNS on ALP activity of gene silencing BMSCs

According to the table above(Table. 2), the activity of ALP in 100 mg group significantly increased by comparing with that of other groups in each introduction time(**P < 0.01); the ALP activity level in 100 mg RNAi group was higher than that of control group(*P < 0.05), especially on day 3(**P < 0.01), which might indicate that PNS had a promoting effect on osteogenesis of TGF-β1 gene silencing cells; the ALP activity level of 100 mg RNAi group was lower than that of 100 mg group, and there was a significant difference in the day 5 and day 7(△*P* < 0.05), while the ALP level of 100 mg RNAi group was higher than that of RNAi group, and a significant difference was found in all three induction time (□*P* < 0.01); comparing with the normal group, there was no statistical significance of the effect on intracellular ALP level among normal group, blank virus group and RNAi group.

**Table 2:** Intracellular ALP activity after RNAi in all groups $\left(\overline{x},\pm, s,,,n,=4\right)$

Group	3 days	5 days	7 days
Normal group	0.490 ± 0.095	1.013 ± 0.334	1.625 ± 0.241
100 mg group	1.031 ± 0.197**	1.675 ± 0.188**	2.578 ± 0.281**
Blank virus group	0.388 ± 0.073	0.868 ± 0.260	1.543 ± 0.329
RNAi group	0.358 ± 0.093	0.833 ± 0.079	1.413 ± 0.156
100 mg RNAi group	0.916 ± 0.214**^□^	1.295 ± 0.103*^△□^	2.144 ± 0.255*^△□^

*: *p*<0.05, other 4 groups compare to Normal group

**: *p*<0.01, other 4 groups compare to Normal group

△: *p*<0.05, 100mg RNAi group compare to 100mg group

□: *p*<0.01, 100mg RNAi group compare to RNAi group

### The effect of PNS on formation of calcium nodes of TGF-β1 gene silencing BMSCs by Alizarin red staining

**Fig. 13:**
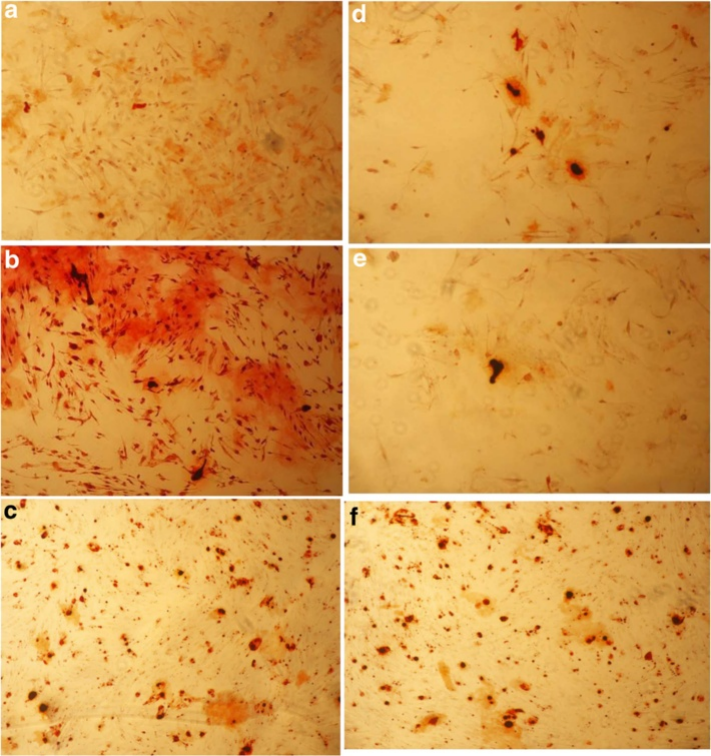
Result of Alizarin red staining of five groups (**a**. normal group, **b**. positive group, **c**. 100 mg group, **d**. blank virus group, **e**. RNAi group, **f**. 100 mg RNAi group)

After 21-day induced cultivation, it was observed that there were some white bulges(nodes) with different densities on the bottom of culture plate when the culture solution was absorbed by those induced cells. After the alizarin red staining, it was showed that calcium nodes turned to be orange or dark red, and the counting results of calcium nodes in each group presented as following (Fig. 13).

According to the table above (Table 3), there were several calcium nodes in 100 mg group without silencing after induction, which had a significant difference with the comparison of the normal group(**P < 0.01); there was no significant difference of the calcium nodes count among the blank virus group, the RNAi group (without induction factors) and the normal group(*P* > 0.05); however, There were also some calcium nodes in 100 mg RNAi group but it was less than that in 100 mg group, which had a significant difference(▲*P* < 0.01). It was showed that gene silencing had an inhibitory effect on induced-osteogenic effects of PNS in a certain degree.

**Table 3:** Comparison of calcium nodule count in each group $\left(\overline{x}\pm s\right)$

Groups	*n*	Calcium nodes count
Normal group	8	2.63 ± 0.52
Positive group	8	120.88 ± 13.80**
100 mg group	8	97.63 ± 12.14**
Blank virus group	8	2.38 ± 0.52
RNAi group	8	3.63 ± 1.41
100 mg RNAi group	8	65.38 ± 11.17**^▲^

**: *p*<0.01, other 5 groups compare to Normal group

▲: *p*<0.01, 100mg RNAi group compare to 100mg group

## Discussion

Bone mesenchymal stem cells(BMSCs) is a bone marrow-derived mutipotent stem cells, which can differentiate into several somatic cells under a certain circumstances, such as: osteoblasts, chondroblasts, fibroblasts, adipocytes and endothelial cells [11]. Since BMSCs are easily obtained, possess multiple differentiate ability and strong proliferative ability, and has a positive response to various bone growth factors, they has been considered as an important seed cells in bone tissue engineering [12]. The Percoll density gradient centrifugation was also applied in the experiment [13, 14]. The high purity of BMSCs was achieved, and its rapid growth was in accordance with the typical characteristic of BMSCs [15]. With reference to the surface markers of BMSCs reported in previous studies [16, 17], in our study, the expression of surface markers of rabbit BMSCs, such as CD29, CD44, CD105, CD61 and CD105, was positive, while those of hematopoietic progenitor cells, such as CD34, CD45, CD11a and HLA-DR, was negative. Our study focused on the detection of three surface markers, including CD29, CD45 and HLA-DR. It was found that CD45 and HLA-DR were negative, while CD29 was positive, which was in accordance with literature reports.

As a cytokine for regulating bone reconstruction, TGF-β1 plays an important role in fracture healing, new bone regeneration as well as the balance between resorption and formation during bone reconstruction. It was reported [18, 19] that there were five positive effects on regulating osteogenesis of TGF-β1, including the transformation of mesenchymal cells, the differentiation of osteoblasts and chondroblasts, the formation and excretion of extracellular matrix, the repairing and reconstruction of bone tissues via the regulation of osteoclasts, as well as the regulation of other hormones and growth factors. TGF-β1 was likely to be the target of osteogenesis-promoting effect for most drugs.

It was reported that PNS with a certain concentration had a promoting effect on osteogenic differentiation of BMSCs of rats and rabbits in vitro, which was related to the down-regulation of expressions of RANKL, reduction of formations and activity of osteoclasts [20], as well as restriction of adipogenic differentiation of BMSCs. In this study, it was found that a certain dosage of PNS had an up-regulating effect on the gene expression of TGF-β1, which suggested that the osteogenesis of PNS was likely related to the up-regulation of gene expression of TGF-β1. Our study focused on not only whether PNS still had osteogenic effects on RNAi cells, but the relationship between PNS and the expression of TGF-β1. RNA interference (RNAi) can transfect target gene-homologous small interferencing RNA(siRNA) into target cells in a certain way. After that, the specific mRNA which had the homologous sequence in target cells was induced to degrade, so as to restraint the protein translation process of target genes. It is similar to the gene knockout but the gene is not to be removed forever, so it is also called as “gene silencing”. The greatest advantage of RNAi is the blockage of both transmission pathway of cell signalings and target spots by aiming at a specific factor. In this experiment, siRNA-carried recombinant pLVX-TGF-β1 shRNA-ZsGreen plasmids was established and packed as lentivirus. Then, the lentivirus was applied to infect target cell BMSCs, which made the siRNA to express in BMSCs and degrade the TGF-β1mRNA. As a result, the gene silencing of TGF-β1 was achieved in BMSCs. The stability of silencing effect was verified via FQ-PCR and Western blot.

Osteoblasts can secrete alkaline phosphatase(ALP), and the activity of ALP elevates when the activity of osteoblasts enhances. High expression of ALP is the early marker of differentiation and maturation of osteoblasts [21], and the deposition of calcium salt and formation of calcium nodes can manifest the further differentiation and maturation of osteoblasts [22]. Therefore, the observation of the drug effect on activity of ALP and mineralization of osteoblasts is an essential approach for the research on osteogenetic effect of drugs. In this study, the blank virus group and the pure RNAi alone group were designed to eliminate the interruption of results caused by virus and gene RNAi. According to the results of intracellular ALP activity detection, the activity of ALP in 100 mg group was higher than that in the normal group, which indicated that PNS might promote the osteogenesis of osteoblasts; the activity of ALP in 100 mg RNAi group was higher than that in normal group but lower than that in 100 mg group, which indicated that PNS might still promote the osteogenesis of osteoblasts after TGFβ1 silencing but the osteogenic potential decreased significantly; and no significant differences of intracellular ALP level was found among normal group, blank virus group and RNAi group. From the results of Alizarin red staining, calcium nodes was rare in the normal group, which suggested that only few cells differentiated into osteblasts. Moreover, the results also showed that the cultured BMSCs had a poor ability in osteogenic differentiation, which was in accordance with the feature of multi-directional differentiation potential of BMSCs. The number of calcium nodes was few in both blank virus group and RNAi group, and it was no significant difference by comparing with the control group. This was also showed that both blank virus and gene silencing could not promote the osteogenic differentiation of BMSCs. The number of calcium nodes in the 100 mg RNAi group was more than that in normal group but fewer than that in 100 mg group. This results might suggest that TGF-β1 RNAi has an inhibitory effect on osteogenic differentiation potential of PNS, and further indicate that the osteogenic potential of PNS may partly mediated by TGFβ1, and was related to the mechanism of TGF-β signaling pathway. However, the inhibitory effect of gene silencing on osteogenic differentiation could not reduce the number of calcium nodes to the level of normal cells and silencing cells. Thus, this suggests that PNS had a promoting effect on osteogenesis in a certain degree, and PNS might exert its osteogenic effect via other cytokines and other signaling pathways, such as bone morphogenetic proteins(BMPs) act upstream and Smads signaling act downstream. The mechanism might be related to various monomer compositions contained in PNS. However, its efficacy was extensive and uncertain because the specific function of each composition was not revealed entirely. In addition, the mechanism of osteogenesis might involve in several cell signal pathways, including TGF-β pathway, Notch pathway [23, 24], Wnt pathway [25, 26], Hedgehog pathway [27] and MAPK pathway [28]. Further research will be launched to identify which pathway and target plays a dominant role in promoting osteogenesis.

## Conclusion

In summary, in this study we found that silencing TGF-β1 gene could interrupt the osteogenic effect of PNS. And we suggest that PNS may have a promoting effect on osteogenic differentiation of rabbits’ BMSCs in vitro by up-regulating the gene expression of TGF-β1. However, further studies, including in vitro and in vivo studies, should be conducted to better verify the effect on osteogenic differentiation of PNS.

## Abbreviations

ALP: Alkaline phosphatase
BMPs: Bone morphogenetic proteins
BMSCs: Bone marrow mesenchymal stem cells
FBS: Fetal bovine serum
MTT: Methylthiazoletetrazolium
PNS: Panax notoginseng saponins
RNAi: RNA interference
TGF-β1: Transforming growth factor beta 1

## References

[1] Gao XM (2007). Chinese pharmacy.

[2] Dong TT, Cui XM, Song ZH, Zhao KJ, Ji ZN, Lo CK, Tsim KW (2003). Chemical assessment of roots of Panax notoginseng in China: regional and seasonal variations in its active constituents. J Agric Food Chem.

[3] Uzayisenga R, Ayeka PA, Wang Y (2014). Anti-diabetic potential of Panax notoginseng saponins (PNS): a review. Phytother Res.

[4] Yang X, Xiong X, Wang J (2014). Sanqi panax notoginseng injection for angina pectoris. Evid Based Complement Alternat Med.

[5] Liu N, Shan D, Li Y, Chen H, Gao Y, Huang Y (2015). Panax notoginseng saponins attenuate phenotype switching of vascular smooth muscle cells induced by Notch3 silencing. Evid Based Complement Alternat Med.

[6] Wen XD, Wang CZ, Yu C, Zhao L, Zhang Z, Matin A, Wang Y, Li P, Xiao SY, Du W, He TC, Yuan CS (2014). Panax notoginseng attenuates experimental colitis in the azoxymethane/dextran sulfate sodium mouse model. Phytother Res.

[7] Yang J, Ren T, Li XD, Qiao ZG (2010). The experiment study of total saponins of Panax notoginseng on promoting fracture healing. J. Med. Res..

[8] Li XD, Wang JS, Chang B, Chen B, Guo C, Hou GQ, Huang DY, Du SX (2011). Panax notoginseng saponins promotes proliferation and osteogenic differentiation of rat bone marrow stromal cells. J Ethnopharmacol.

[9] Liu Y, Hao F, Zhang H, Cao D, Lu X, Li X (2013). Panax notoginseng saponins promote endothelial progenitor cell mobilization and attenuate atherosclerotic lesions in apolipoprotein E knockout mice. Cell Physiol Biochem.

[10] Du WX, Duan SF, Yu XL, Yin LM (2015). Panax notoginseng saponins suppress radiation-induced osteoporosis by regulating bone formation and resorption. Phytomedicine.

[11] Bianco P, Rininucci M, Gronthos S, Robey PG (2001). Bone marrow stromal stem cells: nature, biology, and potential applications. Stem Cells.

[12] Matsumura N, Ochi K, Ichimura M, Mizushima T, Harada H, Harada M (2001). Study on free radicals and panceaticibrosis, pancereatic fibrosis induced by repeated injections of superoxide dismutase inhibitor. Oanceras.

[13] Ma L, Liu DJ, Li DT, Liu PT (2008). Effects of different separate methods and culture conditions on growth proliferation and biological characteristics of rabbit bone marrow mesenchymal stem cells. J Clin Rehabil Tissue Eng Res.

[14] Li X, Zhang Y, Qi G (2013). Evaluation of isolation methods and culture conditions for rat bone marrow mesenchymal stem cells. Cytotechnology.

[15] Friedenstein AJ, Chailakhyan RK, Gerasimov UV (1987). Bone marrow osteogenic stem cells: in vitro cultivation and transplantation in diffusion chambers. Cell T issue Kinet.

[16] Wang XF, Tong PJ, Jin HT (2011). Approaches to isolate, culture and identify the rabbit marrow mesenchymal stem cells. J Tradit Chinese Orthop Traum.

[17] Liu Q, Shi Y, Wang H (2013). Experimental study on isolation, culture and identify of rabbit bone marrow-derived mesenchymal stem cells in vitro. J Tissue Eng Reconstr Surg.

[18] Erlebacher A, Filvaroff EH, Ye JQ, Derynck R (1998). Osteoblastic responses to TGF-β during bone remodeling. Mol Biol Cell.

[19] Schuldiner M, Yanuka O, Itskovitz-Eldor J, Melton DA, Benvenisty N (2000). Effects of eight growth factors on the differentiation of cells derived from human embryonic stem cells. Proc Natl Acad Sci U S A.

[20] Wang DW, Pan H, Li HB, Wang HX, Chen YP (2008). Influence of total saponins of Panax Notoginseng on the alcohol-induced differentiation of bone marrow stromal stem cells. J Clin Rehabil Tissue Eng Res.

[21] Yao XM, Cheng Y, Fang F (2007). Experimental study on YIGUTANG containing serum on osteoblast proliferation and ALP expression. J Zhejiang University Tradit Chinese Med.

[22] Maniatopoulos C, Sodek J, Melcher AH (1998). Bone formation in vitro by stromal cells obtained from bone marrow of young adult rats. Cell Tissue Res.

[23] Hilton MJ, Tu X, Wu X, Bai S, Zhao H, Kobayashi T, Kronenberg HM, Teitelbaum SL, Ross FP, Kopan R, Long F (2008). Notch signaling maintains bone marrow mesenchymal progenitors by suppressing osteoblast differentiation. Nat Med.

[24] Dishowitz MI, Mutyaba PL, Takacs JD, Barr AM, Engiles JB, Ahn J, Hankenson KD (2013). Systemic inhibition of canonical Notch signaling results in sustained callus inflammation and alters multiple phases of fracture healing. PLoS One.

[25] Lin GL, Hankenson KD (2011). Integration of BMP, Wnt, and Notch signaling pathways in osteoblast differentiation. J Cell Biochem.

[26] Chen B, Li XD, Liu DX, Wang H, Xie P, Liu ZY, Hou GQ, Chang B, Du SX (2012). Canonical Wnt signaling is required for Panax Notoginseng Saponin-mediated attenuation of the RANKL/OPG ratio in bone marrow stromal cells during osteogenic differentiation. Phytomedicine.

[27] Plaisant M, Fontaine C, Cousin W, Rochet N, Dani C, Peraldi P (2009). Activation of Hedgehog signaling inhibits osteoblast differentiation of human mesenchymal stem cells. Stem Cells.

[28] Hu Y, Chan E, Wang SX, Li B (2003). Activation of p38 mitogen-activated protein kinase is required for osteoblast differentiation. Endocrinology.

